# Combination IETA Ultrasonographic Characteristics Simple Scoring Method With Tumor Biomarkers Effectively Improves the Differentiation Ability of Benign and Malignant Lesions in Endometrium and Uterine Cavity

**DOI:** 10.3389/fonc.2021.605847

**Published:** 2021-08-30

**Authors:** Dongmei Lin, Liang Zhao, Yunxiao Zhu, Yujun Huang, Kun Yuan, Wenfen Liu, Shengli Li, Xia Guo, Yi Hao

**Affiliations:** ^1^Department of Medical Ultrasonics, South China Hospital of Shenzhen University, Shenzhen, China; ^2^Department of Medical Ultrasonics, Shenzhen Hospital, Southern Medical University, Shenzhen, China; ^3^The Third Affliated Hospital, Southern Medical University, Guangzhou, China; ^4^Department of Medical Ultrasonics, The Seventh Affiliated Hospital of Sun Yat-sen University, Shenzhen, China; ^5^Department of Medical Ultrasonics, Shenzhen Maternity and Child Healthcare Hospital, Shenzhen, China; ^6^Shenzhen Key Laboratory of Viral Oncology, Center for Clinical Research and Innovation (CCRI), Shenzhen Hospital, Southern Medical University, Shenzhen, China

**Keywords:** International Endometrial Tumor Analysis, simple scoring, tumor biomarkers, endometrial cancer/carcinoma, endometrium lesion, uterine cavity lesions

## Abstract

**Objectives:**

To evaluate International Endometrial Tumor Analysis (IETA) ultrasonographic characteristics simple scoring method and tumor biomarkers for the diagnosis of uterine cavity and endometrial lesions.

**Methods:**

We classified and scored the normalized description of IETA ultrasonic characteristics, according to IETA expert consensus literature, previous IETA-related research articles, and the previous research experience of this project group. We conducted a retrospective analysis of the ultrasound images of 594 patients enrolled from January 2017 to June 2020, scored them item by item, and finally calculated the total score of each case. Meanwhile, we combined the results of seven tumor biomarkers. The objective was to evaluate the sensitivity, specificity, coincidence rate, and the area under receiver operating characteristic (ROC) curve of IETA ultrasonographic characteristics simple scoring method and tumor biomarkers for benign and malignant uterine cavity or endometrial lesions. The diagnostic efficiency between the combined method and the single method was compared.

**Results:**

A total of 594 cases were confirmed by postoperative pathology or surgery records, including 475 benign lesions and 119 malignant lesions. In the simple ultrasound scoring method, the average score of benign lesions was 3.879 ± 1.279 and that of malignant lesions was 9.676 ± 4.491. If ≥6.5 points was taken as the cutoff value for the judgment of malignant lesions, the sensitivity, specificity, coincidence rate, and the area under receiver operating characteristic (ROC) curve (AUC) were 76.5%, 96.0%, 92.1%, and 0.935, respectively. The difference in tumor antigen 19-9 (CA19-9) and human epididymal protein 4 (HE4) between benign and malignant lesions was statistically significant (all *p* ≤ 0.01). The other five tumor biomarkers (CA125, CA15-3, SCC-Ag, AFP, and CEA) showed no statistically significant difference in benign and malignant lesions. If the value of CA19-9 ≥13.96 U/ml was taken as cutoff value, the sensitivity, specificity, and coincidence rate of the diagnosis of endometrial benign and malignant lesions were 54.8%, 74.7%, and 70.7%, respectively, and the AUC was 0.620. If the value of HE4 ≥ 39.075 pmol/L was taken as cutoff point, the sensitivity, specificity, coincidence rate, and AUC were 77.4%, 67.9%, 69.8%, and 0.796, respectively. The sensitivity was increased to 97.6% and the AUC was 0.939 when IETA ultrasound characteristics simple scoring method combined CA19-9 and HE4 in parallel test.

**Conclusions:**

In IETA ultrasound characteristics simple scoring method, with ≥6.5 points as the cutoff value, it could quickly and accurately assess the benign and malignant in uterine cavity and endometrial lesions, with high diagnostic value. The diagnostic efficacy of seven tumor biomarkers was all mediocre. Combining with these two methods, the comprehensive diagnosis could improve sensitivity and accuracy and reduce the risk of missed diagnosis.

## Introduction

Endometrial lesions are common gynecological diseases, which have a great impact on women’s physical and mental health and quality of life. In particular, endometrial cancer is one of the most common gynecological malignancies (1–5) and accounts for 20%–30% of malignant tumors of the female reproductive tract (6). The prognosis of early endometrial cancer patients is generally good, with a 5-year survival rate of >90% for Stage IA disease (7–9). However, the prognosis is poor for patients with high-risk endometrial cancer (Grade 3 or non-endometrioid histotype and/or Stage ≥IB) because they are at increased risk of lymph node metastasis, distant tumor spread, and tumor recurrence (7, 9, 10).

Women with early-stage endometrial cancer often have no special clinical symptoms. More detailed uterine examination could make an early diagnosis, which is of great significance to the prognosis, improving the cure and survival rates of the patients. It could relieve the suffering of patients.

The traditional diagnostic methods are diagnostic curettage or hysteroscopy, but both are invasive and cannot accurately reflect the breadth and depth of the lesion (11). Although MRI can be used clinically to distinguish benign from malignant endometrial lesions and assess the degree of invasion (1), it is not widely available clinically due to its high price and long examination time. Transvaginal ultrasonography, which is the most convenient and does no harm to patients, is now widely used (12).

There were a large number of articles about endometrial diseases, but the level of the literature was uneven, and the description of ultrasonic characteristics was varied, without a unified consensus. It not only confused clinicians in interpreting ultrasound reports but also did harm to the development of these fields. Based on the above situation, the experts of the International Endometrial Tumor Analysis group (IETA) have written three normative descriptions of endometrial lesions and consensus literatures related to histopathology from 2010 to 2020 (9, 10, 13).

In the past decades, there were several studies (14–23) on the clinical application of IETA expert consensus, with mixed praises and criticisms on its application effects. Among them, it was criticized that there were too many descriptive indicators of IETA ultrasound characteristics, which took a long time, and the consistency among different observers was poor (22).

Serum tumor biomarkers have been widely used in clinic, but there is no serum tumor biomarker with high specificity for endometrial cancer (3, 24, 25).The carbohydrate antigen 125 (CA125) has been proven to be a useful method in the diagnosis and follow-up of gynecological malignancies (26). It is highly expressed in the serum of patients with malignant tumors but may be increased in varying degrees in the serum of benign lesions or healthy patients. HE4 has been recently identified as a potential biomarker in endometrial carcinoma (3, 4, 27, 28). Some studies (3, 5, 29–33) showed that CA125 and HE4 were significantly correlated with histological grade, stage, lymph node metastasis, muscular invasion, and cervical involvement.

The carbohydrate antigen 15-3 (CA15-3), which is the important specific tumor biomarker for breast cancer (34), has also been found to be highly expressed in endometrial cancer, gastric cancer, and other malignant tumors in recent years (35). The carcinoembryonic antigen (CEA) is one of the markers associated with the stage of tumor development; the diagnostic value of the marker is particularly important in patients with colon and rectum tumors. Some studies have shown that an increase in the level of the CEA antigen in serum was found in a small group of endometrial cancer cases (25).

CA19-9 is an oligosaccharide-related antigen, which is closely related to digestive tract tumors such as pancreatic cancer and gastric cancer, and gynecological tumors. The alpha-fetoprotein (AFP) is an acidic glycoprotein synthesized from yolk sac and liver cells in embryo and is currently the most sensitive marker for the diagnosis of primary liver cancer. The correlation between AFP and endometrial cancer is still at the exploratory stage. Some studies have shown that the expression of AFP in endometrial carcinomas or carcinosarcomas is not uncommon (36).

The squamous cell carcinoma antigen (SCC-Ag) is a sensitive marker of cervical and vulvar squamous cell carcinoma (37). It is one of the proven biomarkers in serum for tumors, and its concentration is enhanced in most gynecological tumors (38, 39).

The relevant studies (6, 33, 34, 40) had shown that imaging examination combined with tumor biomarker detection could significantly improve the clinical diagnosis rate of tumors. However, the literature rarely reported IETA sonographic features combined tumor biomarkers to comprehensively evaluate benign and malignant uterine cavity or endometrial lesions.

In this study, we converted the normalized description of IETA ultrasound characteristics into a simple scoring method based on the existing IETA-related research results (14–23) and the previous research experience of our project team. Meanwhile, we combined the results of tumor biomarkers to evaluate the diagnostic efficacy of benign and malignant lesions in uterine cavity or endometrium. The aim was to provide a rapid and effective method for the clinical diagnosis of benign and malignant lesions in uterine cavity or endometrium.

## Materials and Methods

This is a retrospective study. A total of 594 patients with uterine cavity or endometrial lesions were enrolled who were admitted to The Seventh Affiliated Hospital of Sun Yat-sen University or Shenzhen Hospital of Southern Medical University from January 2017 to June 2020. The retrospective study has been approved by the Medical Ethics Committee of the two hospitals (KY-2020-030-01 and NYSZYYEC20200029), and the need for informed consent was waived.

The inclusion criteria for this study were as follows:

(1) All patients underwent transvaginal/transrectal ultrasonography within 1 month before surgery.
(2) All cases were examined for seven tumor biomarkers within 1 month before surgery.
(3) The human chorionic gonadotropin (HCG) test was performed to exclude pregnancy-related diseases.
(4) There was no adenomyosis in all cases; the abnormality of the endometrial–myometrial junction caused by adenomyosis was excluded.
(5) The curettage or hysteroscopy or surgical excision was performed in enrolled patients, and the results were confirmed by pathological diagnosis or surgical records.

Exclusion criteria were as follows:

(1) All patients who did not meet the inclusion criteria
(2) For medical ethical reasons, patients who were under legal age (<18 years old)
(3) Patients who previously underwent abdominal surgical interventions, where the uterus has been removed
(4) Patients who were allergic to ultrasound gel
(5) Pregnant or lactating women or patients with a recent history of hormonal medication
(6) Patients who had received radiotherapy, chemotherapy, or hormone therapy preoperatively or had tumors in other organs.

### Instruments and Methods

We used three-dimensional ultrasound instruments (GE Voluson E8 and E10, GE Healthcare, Tiefenbach, Zipf, Austria). All instruments were equipped with high frequency intracavitary probe (RIC5-9-D, 5–9 MHz). All patients underwent transvaginal (or transrectal) ultrasound scan in a lithotomy position with an empty bladder, combined with transabdominal scan if necessary.

Every assessment of the uterus should start with identification of the bladder and the cervix. The position of the uterus was noted and measurements taken. The uterus was scanned in the sagittal plane from cornu to cornu and in the (oblique) transverse plane from the cervix to the fundus. Having established an overview of the whole uterus, the image was magnified to contain only the uterine corpus. The magnification should be as large as possible, focusing on the area of interest (13). The probes collected grayscale images and color Doppler ultrasound or energy Doppler ultrasound images of the uterus and bilateral appendages from left to right in multiple sections, and the images or dynamic videos were stored in the hard disk of the machines or in the ultrasonic workstation.

### The Criteria for IETA Ultrasonographic Characteristics Simple Scoring Method

**Figure 1:**
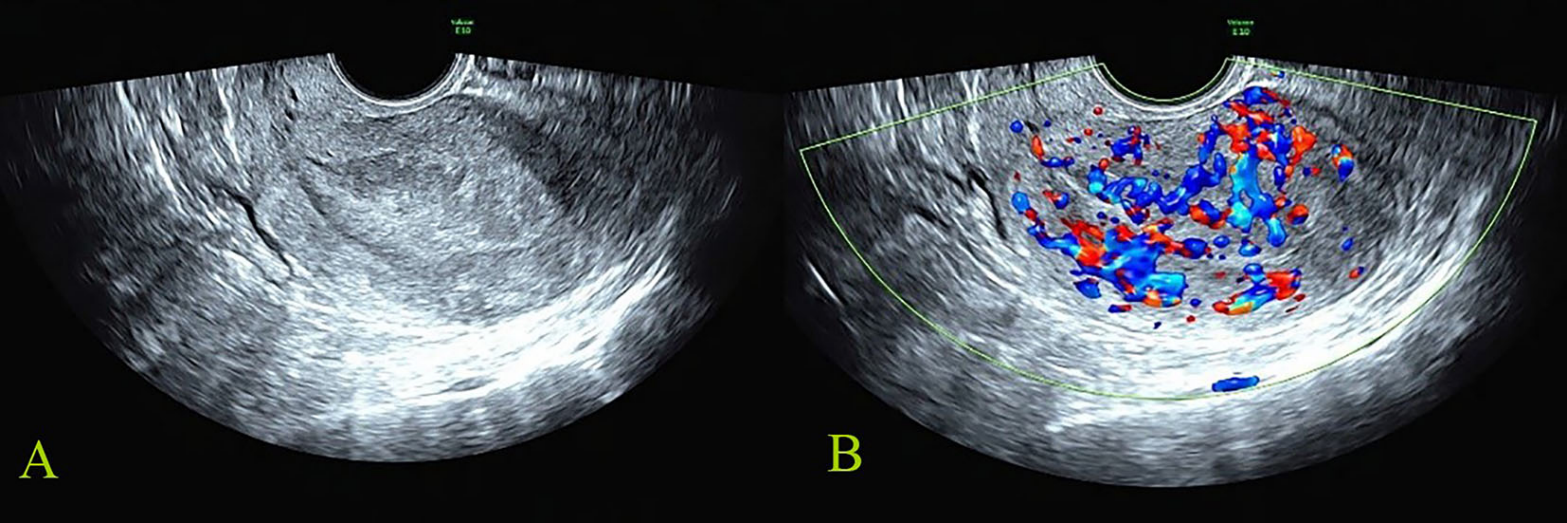
Malignant reference case: female, 56 years old, postmenopause, irregular vaginal bleeding for more than 6 months; CA125, CA19-9, and HE4 were all increased. The postoperative pathology was stage IIIC1 of endometrioid adenocarcinoma. The total score of IETA ultrasound characteristics simple scoring method was 13 points [endometrial thickness 27 mm: 3 points; heterogeneous without cysts: 1 point; interrupted of endometrial–myometrial junction: 2 points; no “Bright edge” sign: 1 point; color score: abundant vascularity 4 points; multiple vessels (multifocal origin) pattern: 2 points]. **(A)** The grayscale image of endometrial lesion; **(B)** the color Doppler flow image of endometrial lesion.

**Figure 2:**
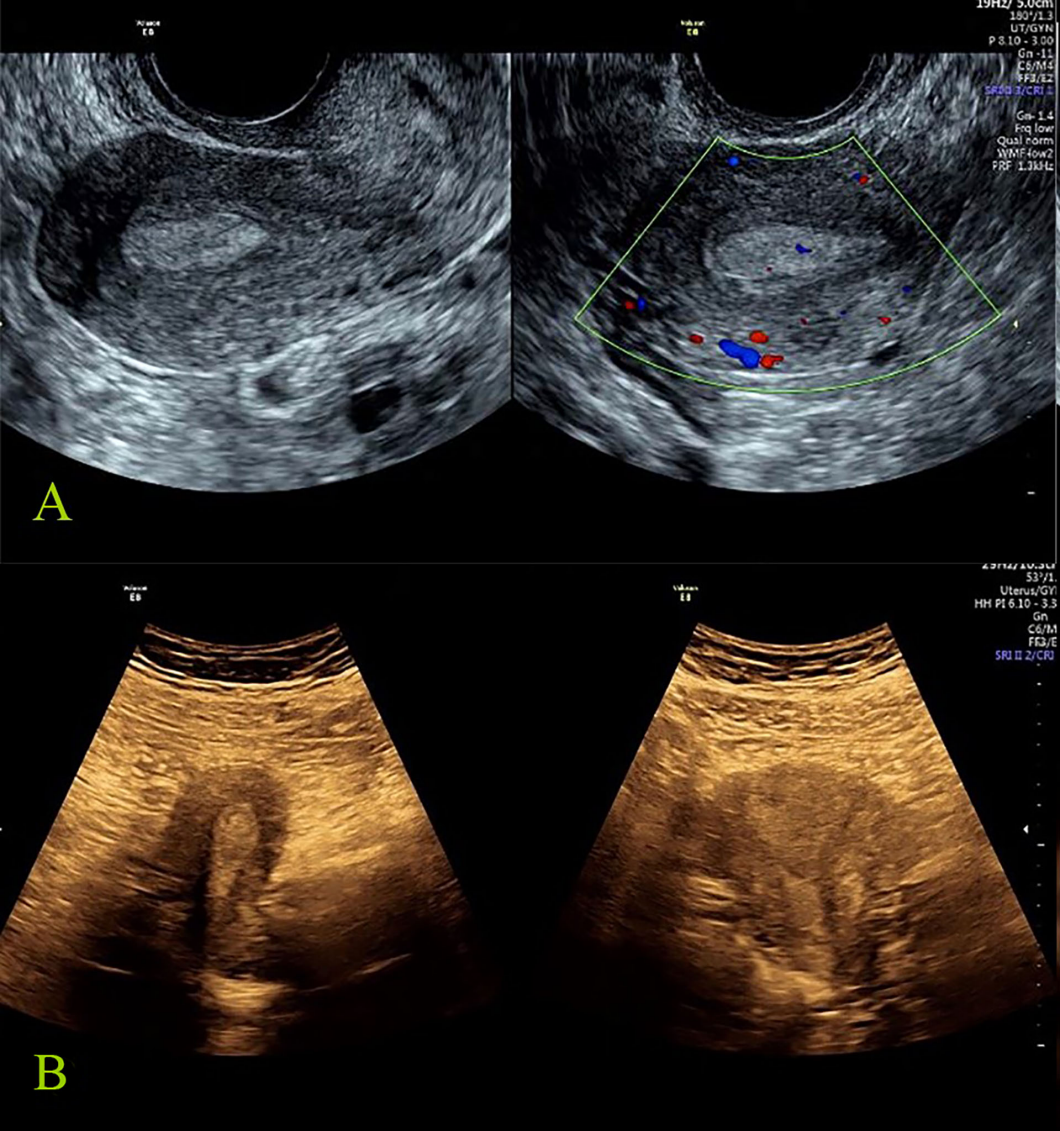
Benign reference case: female, 27 years old, abnormal menstruation for more than 2 months. All the seven tumor biomarkers were normal, the postoperative pathology was endometrial polyp, and the total score of IETA ultrasound characteristics simple scoring method was: 3 points [endometrial thickness 10 mm: 0 point; heterogeneous without cysts: 1 point; regular endometrial–myometrial junction: 0 point; having “Bright edge” sign: 0 point; color score: minimal color/sparse vascularity: 2 point; single vessel (without branching): 0 point]. **(A)** The grayscale and color Doppler ultrasound images of endometrial polyp (transvaginal scan). **(B)** The grayscale images of endometrial polyp (transabdominal scan).

In this study, we classified and scored the normalized description of IETA ultrasonic characteristics, according to IETA expert consensus literature (9, 10, 13), previous IETA-related research literature (14, 15, 18, 19, 22), and the previous research experience of this project group (see **Table 1** for detailed scoring criteria). We conducted a retrospective analysis of the ultrasound images of each patient and scored them item by item and finally calculated the total score of each case. See **Figures 1**, **2** for specific scoring examples.

**Table 1:** The reference table of IETA ultrasound characteristics simple scoring method.

Ultrasound characteristics	Scoring				
	0 point	1 point	2 points	3 points	4 points
Endometrial thickness (mm)					
Premenopause	≤12.0	12.1–15.0	15.1–20.0	>20.1	
Postmenopause	≤5.0	5.1–10.0	10.1–15.0	>15.1	
Echogenicity of endometrium					
Uniform	Homogeneous hyperechoic; Homogeneous hypoechoic; Homogeneous isoechoic; Three-layer pattern;				
Non-uniform	Homogeneous with regular cysts	Homogeneous with irregular cysts; Heterogeneous without cysts; Heterogeneous with regular cysts;	Heterogeneous with irregular cysts		
Endometrial midline appearance	Linear Nonlinear Irregular Not defined				
Endometrial–myometrial junction	Regular	Irregular	Interrupted	Not defined	
“Bright edge”	Yes	No			
Intracavitary fluid	No fluid	Anechogenic or of low-level echogenicity	“Ground glass”	“Mixed” echogenicity	
Color score		no color/no vascularity	minimal color/Sparse vascularity	moderate amount of color/moderate vascularity	abundant color/abundant vascularity
Vascular pattern	No flow; Single vessel (without branching); Single vessel (with branching); Scattered vessels; Circular vessels;	Multiple vessels (focal origin)	Multiple vessels (multifocal origin)		

### Image Analysis

The research coordinator encoded the stored images to mask the personal information. Two senior sonologists who both had more than 10 years working experience in gynecology ultrasound analyzed all the ultrasound images independently without knowing other information after being fully familiar with the specific contents of IETA expert consensus on uterine cavity or endometrial lesions. The two observers scored the ultrasound images characteristics one by one according to the simple scoring diagnostic criteria, and the total score of lesions in each patient was finally calculated. The recorded results of the two observers were summarized. If there was any inconsistent result, the two observers discussed and negotiated, and the negotiated results were analyzed statistically.

### The Detection of Tumor Biomarkers

The results of serum CA125, CA15-3, CA19-9, HE4, SCC-Ag, AFP, and CEA were included.

*Detection Method*: Five milliliters of venous blood of the patients was collected as a test sample and centrifuged at 3,000 rpm for 15 min. The serum was separated, and serums CA125, CA15-3, CA19-9, HE4, SCC-Ag, AFP, and CEA were detected by chemiluminescence. The testing instrument was Abbott I-2000 chemiluminescence instrument, and the reagents were as received.

*Criteria*: Tumor biomarkers were positive when they exceeded the normal value.

*Reference values:* CA l25 ≤ 35 U/ml; CA l5-3 ≤ 35 U/ml; CA l9-9 ≤ 35 U/ml; HE4: premenopause < 70 pmol/L, post-menopause < 140 pmol/L; SCC-Ag ≤ 1.5 ng/ml; AFP ≤ 20 ng/ml; CEA ≤ 5 ng/ml.

### Histopathological Diagnoses

**Figure 3:**
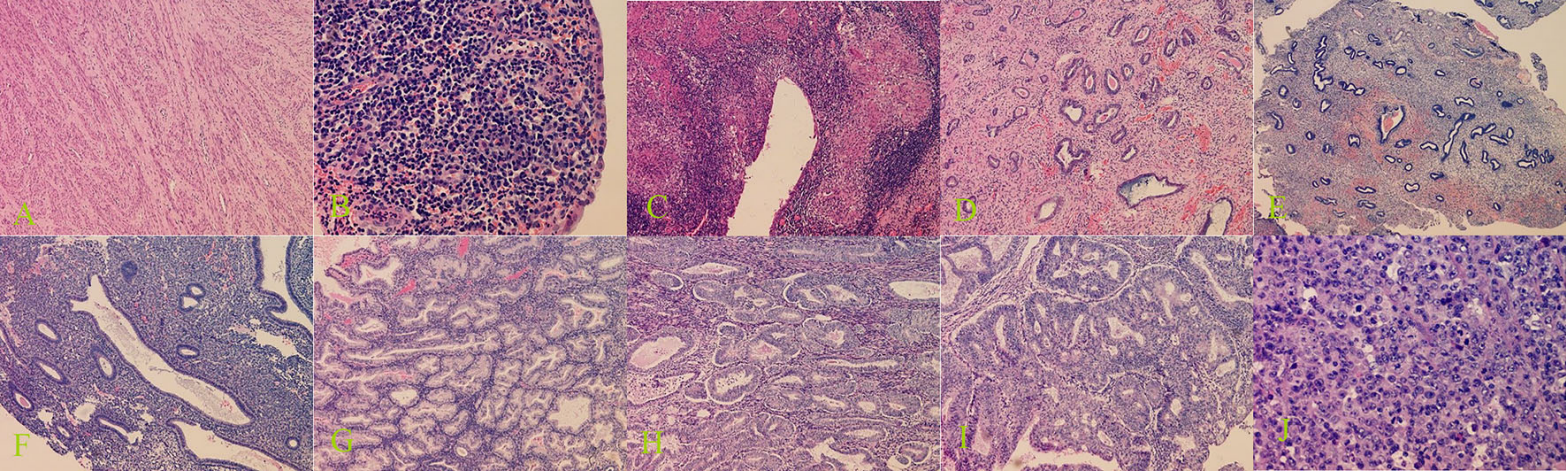
Histopathological examination of benign and malignant lesions of uterine cavity and endometrium. **(A)** Submucous myomas (100× magnification), **(B)** endometritis (400× magnification), **(C)** endometrial tuberculosis (100× magnification), **(D)** endometrial polyps (100× magnification), **(E)** endometrial polypoid hyperplasia (40× magnification), **(F)** endometrial simple hyperplasia (100× magnification), **(G)** endometrial complex hyperplasia (100× magnification), **(H)** endometrial complex hyperplasia with atypical hyperplasia (100× magnification), **(I)** endometrioid adenocarcinoma (100× magnification), and **(J)** uterine undifferentiated sarcoma (400× magnification).

In all cases of intrauterine adhesions, the results were confirmed by surgical records as no pathological specimens were available. The results of other lesions were confirmed by pathological results. Histopathological diagnoses of the specimens were obtained and served as reference standards. All diagnoses were made by specialized gynecological pathologists with more than 10 years’ experience, who were blinded to the results of the ultrasound examination. See **Figure 3** for the characteristics of different pathological types of uterine cavity and endometrial lesions.

### Statistical Analysis

All statistical analyses were performed with SPSS version 23.0 software (IBM Corp. Released 2015. IBM SPSS Statistics for Windows, Version 23.0. Armonk, NY: IBM Corp.). The quantitative data were expressed as mean ± standard deviation. The normal distribution and homogeneity of variance were tested. The independent-samples T-test or nonparametric test was used for the quantitative data. The values of categorical variables were presented by percentage (%); the chi-square test or Fisher’s exact test was used for data comparison.

The ROC curve and the parallel test were used to analyze the sensitivity, specificity, and coincidence rate of simple ultrasound scoring method and simple ultrasound scoring method plus tumor biomarkers in the diagnosis of benign and malignant endometrial lesions; meanwhile, the respective area under the curve (AUC) was obtained. The ROC curve evaluated the diagnostic effectiveness of the value of AUC, which was between 1.0 and 0.5. When the AUC > 0.5, the closer AUC was to 1, the better the diagnostic effect. The AUC > 0.9 was of high diagnostic value. If the AUC was between 0.7 and 0.9, the diagnostic value was moderate. If the AUC was between 0.5 and 0.7, the diagnostic value was low. When AUC = 0.5, it indicated that the diagnostic method was completely ineffective and had no diagnostic value. *p* < 0.05 was considered statistically significant.

## Results

### General Information

There were 594 cases enrolled in this study; 475 cases were benign lesions, and 119 cases were malignant lesions. The endometrial atypical hyperplasia was classified as malignant lesions in this study because the current clinical treatment was based on malignant lesions. See **Table 2** for the specific pathological types.

**Table 2:** Histopathological diagnoses.

Histopathology	Number	%
**Benign lesions**	475	80.0%
Endometrial polyps	226	38.1%
Submucous myomas	42	7.1%
Endometrial simple hyperplasia	57	9.6%
Endometrial complex hyperplasia	15	2.5%
Endometrial polypoid hyperplasia	63	10.6%
Intrauterine adhesions	58	9.8%
Endometritis	12	2.0%
Endometrial tuberculosis	2	0.3%
**Malignant lesions**	119	20.0%
Endometrial hyperplasia with atypia	18	3.0%
Endometrial low grade squamous epithelial lesion	1	0.2%
Uterine giant cell type high-grade undifferentiated Sarcoma	3	0.5%
Endometrioid adenocarcinoma	97	16.3%
FIGO stage IA	58	9.8%
FIGO stage IB	19	3.2%
FIGO stage II	7	1.2%
FIGO stage ≥ III	13	2.2%

The differences between the benign and malignant groups were statistically significant in age, the proportion of patients before and after menopause, body mass index (BMI), the proportion of patients with irregular vaginal bleeding, and the number of deliveries (all *p* < 0.05). The demographic and clinical variables are shown in **Table 3**.

**Table 3:** Demographic and clinical variables of the study group.

Parameter	Benign lesions	Malignant lesions	*p*-value
Cases number (n)	475	119	
Premenopause (%)	432/475 (90.9%)	80/119 (67.2%)	0.000*
Postmenopause (%)	43/475 (9.1%)	39/119 (32.8%)	0.000*
Age (years, mean ± SD)	38.361 ± 9.535	49.353 ± 11.359	0.000*
BMI (kg/m^2^)	22.527 ± 3.646	24.293 ± 2.935	0.006*
Gravity (mean ± SD)	2.415 ± 1.885	2.727 ± 1.773	0.359
Parity (mean ± SD)	1.356 ± 1.083	1.879 ± 1.341	0.036*
Abortion (mean ± SD)	1.051 ± 1.367	0.848 ± 1.093	0.408
Clinical symptoms			
Irregular bleeding of the vagina (%)	107/475 (22.5%)	67/119 (56.3%)	0.000*
Irregular menstruation (%)	256/475 (53.9%)	63/119 (52.9%)	0.816
Hypogastralgia (%)	42/475 (8.8%)	17/119 (14.3%)	0.207
Leucorrhea with blood or contact bleeding (%)	13/475 (2.7%)	3/119 (2.5%)	1.000
No symptom (%)	128/475 (27.0%)	14/119 (11.8%)	0.09

BMI, body mass index [BMI = weight (kg)/height 2 (m)]; mean ± SD, mean ± standard deviation.

*Represents statistical difference between display rates (p < 0.05).

### The Correlation Between IETA Ultrasonic Characteristics and Lesions in Uterine Cavity or Endometrium

The differences between benign and malignant lesions in endometrial thickness, endometrial echogenicity, endometrial midline appearance, interrupted or not defined endometrial–myometrial junction, intracavitary fluid, “bright edge” sign, color score, and vascular pattern were statistically significant (all *p* < 0.05) (**Table 4**).

**Table 4:** The IETA ultrasonographic characteristic comparison of benign and malignant lesions in uterine cavity or endometrium.

Comparison items	Benign lesions	Malignant lesions	*p*-value
Endometrial thickness (mm)	9.883 ± 4.169	15.156 ± 7.353	0.000*
Premenopause (mean ± SD)	10.033 ± 4.076	13.955 ± 6.543	0.011*
Postmenopause (mean ± SD)	8.600 ± 4.969	17.800 ± 8.664	0.009*
Uniform endometrial echogenicity (%)	31/475 (6.5%)	0/119 (0.0%)	
Non-uniform endometrial echogenicity (%)	444/475 (93.5%)	119/119 (100%)	
Endometrial midline appearance			0.000*
Linear (%)	153/475 (32.2%)	7/119 (5.9%)	
Non-linear (%)	79/475 (16.6%)	10//119 (8.4%)	
Irregular (%)	114/475 (24.0%)	21/119 (17.6%)	
Not defined (%)	129/475 (27.2%)	81/119 (68.1%)	
Endometrial – myometrial junction			0.000*
Regular (%)	443/475 (93.2%)	43/119 (36.1%)	
Irregular (%)	5/475 (1.1%)	6/119 (5.1%)	
Interrupted (%)	18/475 (3.8%)	45/119 (37.8%)	
Not defined (%)	9/475 (1.9%)	25/119 (21.0%)	
The ratio of having “bright edge” (%)	183/475 (38.5%)	4/119 (3.4%)	0.000*
Intracavitary fluid			0.000*
No fluid (%)	434/475 (91.4%)	84/119 (70.6%)	
Anechogenic or of low-level echogenicity (%)	35/475 (7.4%)	3/119 (2.5%)	
“Ground glass” (%)	4/475 (0.8%)	11/119 (9.2%)	
“Mixed” echogenicity (%)	2/475 (0.4%)	21/119 (17.7%)	
Color score (mean ± SD)	1.72 ± 0.494	2.84 ± 0.808	0.000*
Vascular pattern			0.000*
No flow	139/475 (29.3%)	0/119 (0.0%)	
Single vessel (without branching) (%)	178/475 (37.5%)	5/119 (4.2%)	
Single vessel (with branching) (%)	18/475 (3.8%)	12/119 (10.1%)	
Scattered vessels (%)	105/475 (22.1%)	18/119 (15.1%)	
Circular vessels (%)	30/475 (6.3%)	0/119 (0.0%)	
Multiple vessels (focal origin) (%)	1/475 (0.2%)	21/119 (17.7%)	
Multiple vessels (multifocal origin) (%)	4/475 (0.8%)	63/119 (52.9%)	
The summary of scores by simple scoring method (mean ± SD)	3.879 ± 1.279	9.676 ± 4.491	0.000*

mean ± SD, mean ± standard deviation.

*Represents statistical difference between display rates (p < 0.05).

The cases with uniform endometrial echogenicity were 100% benign lesions. Almost 100% of malignant cases presented with non-uniform endometrial echogenicity. Of the cases with “bright edge” signs, 97.9% (183/187) were benign. The blood flow in malignant lesions (color score: 2.84 ± 0.808) was significantly higher than that in benign lesions (1.72 ± 0.494).

The vascular pattern of patients with endometrioid adenocarcinoma or other endometrial malignant lesions was dominated by multiple vessels (focal origin or multifocal origin) vascular pattern, accounting for about 94.4% (84/89). The endometrial vessels in patients with endometrial atypical hyperplasia were varied, 27.8% (5/18) were multiple vessels pattern, 33.3% (6/18) were single vessel pattern, and 38.9% (7/18) were scattered vessels pattern. The endometrial hyperplasia (except atypical hyperplasia) was mainly of scattered vessels pattern (62.8%), the endometrial polyps were mainly of single vessel pattern (73.7%), and the submucous myomas were mainly of circular vessels pattern (77.1%).

### Evaluation of IETA Ultrasound Characteristics Simple Scoring Method in the Diagnosis of Benign and Malignant Uterine Cavity or Endometrial Lesions

**Figure 4:**
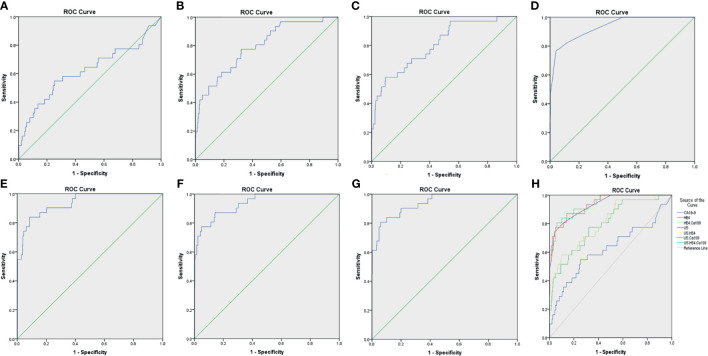
The ROC curves for various diagnostic methods. **(A)** The ROC curve of tumor biomarker CA19-9. **(B)** The ROC curve of tumor biomarker HE4. **(C)** The ROC curve of tumor biomarkers HE4 combined with CA19-9. **(D)** The ROC curve of US. **(E)** The ROC curve of US combined with tumor biomarker HE4. **(F)** The ROC curve of US combined with tumor biomarker CA19-9. **(G)** The ROC curve of US combined with tumor biomarkers HE4 and CA19-9. **(H)** The ROC curves for various diagnostic methods, US, IETA ultrasound characteristics simple scoring method; ROC, receiver operating characteristic curves.

In the IETA ultrasound characteristics simple ultrasound scoring method, the average score of benign lesions was 3.879 ± 1.279, and the average score of malignant lesions was 9.676 ± 4.491. With ≥6.5 points as the cutoff value for the judgment of malignant lesions, the sensitivity, specificity, and coincidence rates were 76.5%, 96.0%, and 92.1%, and the area under ROC curve (AUC) was 0.935, as shown in **Figure 4**.

### The Correlation Between Tumor Biomarkers and Lesions in Uterine Cavity or Endometrium

As shown in **Tables 5**–**7**, tumor biomarkers CA125, CA15-3, SCC-Ag, AFP, and CEA all showed no statistically significant difference in benign and malignant lesions (0.266 ≤ *p* ≤ 1.000). The difference between CA19-9 and HE4 in benign and malignant lesions was statistically significant (all *p* ≤ 0.01).

**Table 5:** A summary table of the specific values and positive rates of seven female tumor markers.

Comparison items	Benign lesions	Malignant lesions	*p*-value
CA125			
mean ± SD (U/ml)	22.500 ± 22.578	27.167 ± 32.742	0.442
positive rate (%)	54/475 (11.4%)	14/119 (11.8%)	0.774
HE4			
mean ± SD (U/ml)	35.816 ± 11.924	249.302 ± 972.927	0.000*
positive rate (%)	5/475 (1.1%)	21/119 (17.6%)	0.000*
CA15-3			
mean ± SD (U/ml)	9.761 ± 10.528	11.551 ± 6.896	0.353
positive rate (%)	3/475 (0.6%)	1/119 (0.8%)	1.000
CA19-9			
mean ± SD (U/ml)	12.358 ± 18.197	31.768 ± 48.387	0.000*
positive rate (%)	24/475 (5.1%)	25/119 (21.1%)	0.002*
CEA			
mean ± SD (U/ml)	1.681 ± 6.622	1.737 ± 0.993	0.962
positive rate (%)	4/475 (0.8%)	3/119 (2.5%)	0.266
SCC-Ag			
mean ± SD (U/ml)	0.927 ± 0.755	1.198 ± 1.787	0.408
positive rate (%)	32/475 (6.7%)	10/119 (8.4%)	0.487
AFP			
mean ± SD (U/ml)	2.985 ± 7.830	2.565 ± 1.357	0.766
positive rate (%)	2/475 (0.4%)	1/119 (0.8%)	1.000

mean ± SD: mean ± standard deviation; CA19-9, carbohydrate antigen 19-9; HE4, human epididymis protein 4; AFP, alpha-fetoprotein; CEA, carcinoembryonic antigen; CA125, carbohydrate antigen 125; CA15-3, carbohydrate antigen 15-3; SCC-Ag, squamous cell carcinoma antigen.

*Represents statistical difference between display rates (p < 0.05).

**Table 6:** Expression of serum molecular markers of carcinoma in benign and malignant endometrium lesions.

Tumor markers	Cutoff value	AUC	SE	95% CI	Sensitivity (%)	Specificity (%)	*p-*value malignant vs. benign
CA19-9	13.96 U/ml	0.620	0.062	0.498,0.743	54.8%	74.7%	0.026*
HE4	39.075 pmol/L	0.796	0.043	0.712,0.880	77.4%	67.9%	0.000*
AFP	–	0.490	0.053	0.385,0.594	71.0%	34.5%	0.846
CEA	–	0.513	0.054	0.412,0.619	88.2%	23.6%	0.663
CA125	–	0.518	0.055	0.411,0.625	32.3%	77.3%	0.740
CA15-3	–	0.600	0.053	0.496,0.705	61.3%	59.3%	0.063
SCC-Ag	–	0.512	0.054	0.407,0.617	90.3%	20.8%	0.617

AUC, the area under the curve; SE, standard error; 95% CI, 95% confidential interval; CA19-9, carbohydrate antigen 19-9; HE4, human epididymis protein 4; AFP, alpha-fetoprotein; CEA, carcinoembryonic antigen; CA125, carbohydrate antigen 125; CA15-3, carbohydrate antigen 15-3; SCC-Ag, squamous cell carcinoma antigen. *represents statistical difference between the two comparison items (P < 0.05).

**Table 7:** The ROC curve analysis values of each diagnostic method and the diagnostic value of parallel test in combined screening (the values in brackets are the diagnostic indicators of parallel tests).

Methods	Cut-off value	AUC	95% CI	Sensitivity (%)	Specificity (%)	Coincidence rate(%)	Missed diagnosis rate (%)	Misdiagnosis rate (%)
CA19-9	13.96 U/ml	0.620	0.498,0.743	54.8%	74.7%	70.7%	45.2%	25.3%
HE4	39.075 pmol/L	0.796	0.712,0.880	77.4%	67.9%	69.8%	22.6%	32.1%
CA19-9+HE4		0.805	0.723,0.888	58.1% (89.8%)	90.4%	83.9%	41.9% (10.2%)	9.6%
US	6.5 points	0.935	0.892,0.977	76.5%	96.0%	92.1%	23.5%	4.0%
US+HE4		0.936	0.894,0.978	83.9% (94.7%)	91.5%	90.0%	16.1% (5.3%)	8.5%
US+CA19-9		0.937	0.895,0.979	87.1% (89.4%)	85.8%	86.1%	12.9% (10.6%)	14.2%
US+HE4+CA19-9		0.939	0.898,0.980	80.6% (97.6%)	94.0%	91.3%	19.4% (2.4%)	6.0%

US, IETA ultrasound characteristics simple scoring method; AUC, the area under the curve; % CI: 95% confidential interval.

With the value of CA19-9 ≥13.96 U/ml as cutoff value, the sensitivity, specificity, and coincidence rate of the diagnosis of benign and malignant endometrial lesions were 54.8%, 74.7%, and 70.7% and AUC of 0.620. With the value of HE4 ≥39.075 pmol/L as truncation value, the sensitivity, specificity, coincidence rate, and AUC of the diagnosis of benign and malignant lesions were 77.4%, 67.9%, 69.8%, and 0.796, respectively.

### The Evaluation of IETA Ultrasound Characteristics Simple Scoring Method Combined Tumor Biomarkers for Benign and Malignant Lesions in Uterine Cavity or Endometrium

When CA19-9 was combined with HE4 in parallel, the diagnostic sensitivity, specificity, and AUC were increased to 89.8%, 90.4%, and 0.805, respectively. As shown in **Table 7**, the sensitivity of IETA ultrasound characteristics simple scoring method combined HE4 examination could be increased to 94.7%, and the AUC was improved slightly (0.936). The sensitivity and AUC of the simple scoring method combined with CA19-9 were improved to 89.4% and 0.937, respectively. The sensitivity and AUC of IETA simple score method combined CA19-9 and HE4 showed an increase of 97.6% (0.939). See **Figure 4** and **Table 7** for details.

## Discussion

Early diagnosis of benign and malignant lesions in uterine cavity or endometrium is of great significance for early clinical treatment and improvement of prognosis (2, 38). The imaging examinations are important methods, especially transvaginal ultrasonography (17).

In this research, we classified and scored the normalized description of IETA ultrasonic characteristics, according to IETA expert consensus literature, previous IETA-related research literature, and the previous research experience of this project group. We found that this simple scoring method could not only rapidly assess the benign and malignant lesions in uterine cavity or endometrium but also had high diagnostic accuracy (AUC, 0.935). Dueholm et al. (15, 18) had developed a Doppler parameter scoring system based on the best performance Doppler parameters. They used multivariate logistic regression to establish a prediction model for endometrial malignancy. Sladkevicius et al. (21) also studied a prospective temporal validation of mathematical models to calculate risk of endometrial malignancy in patients with postmenopausal bleeding. Although these prediction models were also of high diagnostic efficacy, they were both targeted at postmenopausal women with endometrial thickness ≥4.5/5 mm and accompanied by postmenopausal bleeding. The scope of their research was relatively limited and could not be widely promoted. The IETA ultrasound characteristics simple scoring method established in this study was a fast and effective diagnostic method for all pre- and postmenopausal women. It was expected to be clinically applicable. In addition, this study combined the results of serum tumor biomarkers for joint diagnosis. At present, the literature rarely reported IETA ultrasonographic characteristic combined tumor biomarkers to comprehensively evaluate benign and malignant uterine cavity or endometrial lesions.

In the study of Kabil Kucur et al. (14), 100% of endometrial cancer cases had non-uniform endometrium. The endometrial thickness in malignant lesions was significantly thicker than that in benign lesions. Single vessel (with or without branching) pattern was an apparent finding for endometrial polyps. Multiple vessels pattern was seen significantly higher in endometrial cancer. A statistically significant relationship was found between scattered vessel pattern and endometrial hyperplasia. A statistically significant relationship was also found between submucous myomas and circular vessels pattern. Circular vessels pattern was observed only in submucous myomas, not with other endometrial pathologies. Their results above were similar to those of this study. However, in their study, the color score of the endometrium lesions was not statistically different among different endometrial pathologies. But in our study, the difference in color score in benign and malignant lesions was statistically significant (*p* < 0.01). In most benign lesions, the color score was ≤2 points, while, the color score was ≥3 points in most malignant lesions. The results of Alcazar et al. (17) was similar to ours. Meanwhile, Alcazar et al. confirmed that the reproducibility of assigning the IETA color score for assessing endometrial vascularization using three-dimensional volumes was good regardless of the experience of the examiner.

In the research of Dueholm et al. (15), patient’s age, BMI, endometrial thickness, endometrial–myometrial junction, and color score were reported as good indicators to predict endometrial malignant tumors, which were similar to the results of our study. However, in their study, the endometrial vascular pattern had no significant value in predicting malignant lesions, which was contrary to our finding. In our study, the endometrial vascular pattern was significantly correlated with benign and malignant lesions, and the vascular pattern in endometrial malignant lesions was mostly dominated by multiple vessels (focal origin or multifocal origin) pattern.

In this study, none of the 13 women with endometrium <3mm had endometrial cancer (or atypical hyperplasia). Thirty-one cases with uniform endometrial echogenicity (including three-layer pattern) were all benign lesions. Seven of 160 endometria (4.4%) with a linear endometrial midline were malignant lesions, and 2.7% (5/183) cases with a single vessel without branching on unenhanced ultrasound were malignant lesions. Our results above are similar to the research conclusion of Van den Bosch et al. (23) that some easy to assess IETA features (i.e., endometrial thickness <3 mm, triple layer pattern, linear midline, and single vessel without branching) make endometrial cancer unlikely.

In this research, we included the endometrial atypical hyperplasia in the malignant lesion group, based on its clinical treatment protocol, was similar to malignant lesions. If it was removed from the malignant group refering to the study of Sladkevicius et al. (21), the diagnostic sensitivity of IETA ultrasound characteristics simple scoring method was increased to 86.1% (87/101).

In the simple scoring method of this study, we weighted the score according to the degree of correlation between IETA ultrasound characteristics and benign and malignant lesions. Endometrial thickness, endometrial–myometrial junction, “bright edge” sign, intracavitary fluid, color score, and multiple vessels pattern, which were significantly related to benign and malignant lesions, would be given points with greater weight. The indicators that were not significantly associated with the differentiation of benign from malignant may be assigned a less weighted score or no score.

Although the endometrial midline appearance between benign and malignant lesions was statistically significant, the irregular or not defined endometrial midline appearance was also accounted for the majority of benign lesions (51.2%), leading to great difficulties in the identification of benign and malignant lesions. Moreover, in this study, we found that if the score was assigned to the endometrial midline appearance, and ≥9.5 points were used as the cutoff value for the judgment of malignant lesions, the sensitivity, specificity, and area under ROC curve (AUC) were 70.6%, 97.0%, and 0.931; it not only increased the evaluation time but also slightly decreased the diagnostic efficacy. Therefore, in this research, the endometrial midline appearance was not assigned to the score.

In this study, HE4 had the highest diagnostic efficiency; it had higher sensitivity (77.4%) and better diagnostic efficiency (AUC, 0.796) than any other single tumor biomarker. The results were similar to those of Ge et al. (2), Li et al. (27), and Bian et al. (41). The diagnostic performance of CA19-9 was slightly lower. If serum HE4 combined with CA19-9 examination, the diagnostic efficiency could be further increased (sensitivity, 89.8%; specificity, 90.4%; AUC, 0.805).

The other tumor biomarkers (CA125, CA15-3, SCC-Ag, AFP, and CEA) had poor diagnostic efficacy. The overall diagnostic efficacy of serum tumor biomarkers was not high; as Kozakiewicz (25) said, none of them was recognized by the experts as relevant, i.e., sufficiently sensitive and specific in the diagnosis and prognosis of the course of endometrial cancer.

Nithin et al. (35) observed that serum CA125 with reasonable sensitivity and specificity has the best diagnostic utility in differentiating endometrial cancer in patients presenting with abnormal uterine bleeding. However, in our study, the performance of CA125 was not ideal. We analyzed that the possible reasons were as follows: First, CA125 was not only a diagnostic marker for a variety of malignancies but also increased in some benign lesions or healthy individuals and has no significant specificity in endometrial cancer. Second, there were few malignant cases in this study, and most of them were early cancerization. The expression level of CA125 in serum was not high, and there was no significant difference between benign and malignant lesions. In addition, Unsal et al. (33) found that the mean CA125 level in endometrioid-type EC was significantly lower than that in non-endometrioid-type EC. In this study, endometrioid adenocarcinoma accounted for 96% (97/101) of endometrial cancer cases, and the expression level of CA125 was mostly low, which was also one of the reasons for the unsatisfactory diagnostic efficacy of CA125.

### The Analysis of Missed and Misdiagnosed Cases by IETA Ultrasound Characteristics Simple Scoring Method

In this study, the rate of missed diagnosis by the simple scoring method was 23.5% (28/119), and the rate of misdiagnosis was 4.0% (19/475). Among the 28 malignant cases missed, 14 cases were endometrial atypical hyperplasia, and 14 cases were Stage IA endometrioid adenocarcinoma. The possible factors for missed diagnosis were the following: all the missed cases were endometrial atypical hyperplasia or early stage lesions, endometrial thickness was not significantly thickened, there was no obvious invasion of the endometrial–myometrial junction, the lesions’ blood flow was not much, and the vascular pattern was dominated by single vessel or scattered vessels pattern, so the total score was not high and could not be distinguished from benign lesions.

However, among the missed cases, there were 10 cases with CA19-9 value exceeding the threshold (13.96 U/ml), 8 cases with HE4 value exceeding the threshold (39.075 pmol/L), and 7 cases with CA19-9 and HE4 value both exceeding the threshold. Therefore, the sensitivity of the IETA ultrasound simple scoring method combined tumor markers HE4 and CA19-9 could be significantly increased (up to 97.6%), and the rate of missed diagnosis can be reduced.

Among the 19 cases misdiagnosed, there were 11 cases of submucous myomas, 2 cases of endometrial polyp, 3 cases of endometrial polypoid hyperplasia, 1 case of endometrial simple hyperplasia, and 2 case of endometrial complex hyperplasia. The cases of misdiagnosis were mainly submucous myomas, which were endowed with high score in the scoring method due to the interruption of the endometrial – myometrial junction, leading to false positive results. However, the echoes of submucous myomas were mostly hypoechoic, and the vascular pattern was mostly circular vessels pattern, which were significantly different from the echoes of endometrium malignant lesions (mainly hyperechoic or mixed echogenicity) and the vascular pattern of lesions (mainly multiple vessels pattern). Therefore, in practical work, the rate of misdiagnosis could be reduced by combining with other ultrasonic characteristic of the lesions while assigning scores.

There are many advantages of transvaginal ultrasound, such as low cost, clear imaging, non-invasive, convenient, and repeatable operation. The cost of serum tumor biomarkers examination is also relatively low; the results are fast and reliable.The two tests are available in the vast majority of hospitals, including remote ones with poor medical equipments. Although the IETA ultrasound characteristics simple scoring method has better specificity and diagnostic efficiency, its sensitivity is still deficient. The diagnostic efficiency of tumor biomarkers examination for endometrial and uterine cavity lesions is not good. Combined with these two methods, the specificity decreased slightly, but the diagnostic sensitivity can be greatly improved. The prognosis was good for those who were diagnosed with early stage EC, with a 5-year survival rate higher than 90% (7–9), but most of EC patients were detected at medium or advanced stages of the cancer, who often with lymph node or distant metastasis; the prognosis was poor, who may lose the opportunity of surgical therapy (42). For these patients with recurrent or metastatic disease, the median overall survival (OS) remained short, with 5-year overall survival rates as low as 16% (43, 44). In clinical practice, due to the above reasons, we prefer to combine these two technologies, which can significantly improve the clinical detection rate of endometrial malignant lesions, contribute to the early detection and early treatment of patients, and improve the survival rate of patients.

### Limitations

There were several limitations in this research. First, this study was a retrospective study, Thus, there were some biases (selection bias, recall bias, etc.); for example, in the IETA 2010 consensus literature, it was suggested that a sonographic examination should preferably be performed in the early proliferative phase (cycle days 4–6) before the menopause, but we cannot guarantee that all premenopausal patients would be screened at the preferable time. In addition, the patient’s medical history, especially the history of hormone administration, can only be provided through clinical history data or return call. If the patient’s memory is incomplete, its accuracy may be different from the real situation. Second, the number of malignant cases in this study was relatively small, and it is relatively difficult to study the stage and grade of endometrial malignant lesions and histopathological types. In future studies, multicenter large-scale prospective studies are needed to conduct in-depth studies on these aspects.

## Conclusion

In IETA ultrasound characteristics simple scoring method, with ≥6.5 points as the cutoff value, it could quickly and accurately assess the benign and malignant uterine cavity and endometrial lesions, with high diagnostic value. The diagnostic efficacy of seven tumor biomarkers was all moderate. Combining with these two methods, the comprehensive diagnosis could improve sensitivity and accuracy and reduce the risk of missed diagnosis. In low-income countries or areas with poor health systems, they are often unable to purchase expensive medical examination equipments, such as MRI or PET-CT, etc.; the medical equipments there are relatively backward. They are eager for a cheap, quick and effective way to help them with their daily diagnostic work. In our study, the transvaginal ultrasound examination and tumor biomarker examination are relatively simple and inexpensive examinations with high comprehensive diagnostic efficacy. We hope that our comprehensive diagnostic method can be recommend in low-income countries or areas with poor health systems.

## Data Availability Statement

The original contributions presented in the study are included in the article. Further inquiries can be directed to the corresponding authors.

## Ethics Statement

This study has been approved by the Medical Ethics Committee of the Seventh Affiliated Hospital of Sun Yat-sen University and Shenzhen Hospital of Southern Medical University (KY-2020-030-01 and NYSZYYEC20200029). Written informed consent for participation was not required for this study in accordance with the national legislation and the institutional requirements.

## Author Contributions

DL: study concepts and design, literature research, data collection, statistical analysis, manuscript writing. LZ: design work, data collection, statistical analysis. YZ: data collection and statistical analysis. YHu: data collection. KY: the research coordinator. WL: data analysis, data collection; SL: data collection, data analysis and manuscript editing; XG: statistical analysis and manuscript editing. YHa: project development, literature research, data management, and manuscript editing. All authors contributed to the article and approved the submitted version.

## Funding

This study was supported by the general programs from the Natural Science Foundation of Guangdong Province (2021A1515011585); Shenzhen Science and Technology Innovation Committee (grant nos. JCYJ20190814110207603 and JCYJ20190814111801681); the National Natural Science Foundation of China (grant no. 81972423); the clinical research start-up plan of Southern Medical University (Grant no. LC2016YM018); the incubation programme of National Natural Science Foundation of China from Shenzhen Hospital of Southern Medical University (grant no. PY2020ZY02); the social public welfare project of science and Technology from Shenzhen Baoan District (grant no. 2016CX301); the Grant of Shenzhen Key Laboratory of Viral Oncology (ZDSYS201707311140430); and the Grant of Shenzhen Sanming Medical Project (SM201702). The funding bodies had no influence on the study design or the collection, analysis or interpretation of data.

## Conflict of Interest

The authors declare that the research was conducted in the absence of any commercial or financial relationships that could be construed as a potential conflict of interest.

The reviewer HX declared a shared affiliation, with several of the authors DL, YZ, YH, KY, and WL, to the handling editor at the time of the review.

## Publisher’s Note

All claims expressed in this article are solely those of the authors and do not necessarily represent those of their affiliated organizations, or those of the publisher, the editors and the reviewers. Any product that may be evaluated in this article, or claim that may be made by its manufacturer, is not guaranteed or endorsed by the publisher.
